# Digital Health–Based Peer Support Ecosystem for Gestational Diabetes Mellitus in Vietnam (VALID II Study): Multistakeholder Cocreation and Pilot Study

**DOI:** 10.2196/82434

**Published:** 2026-05-06

**Authors:** Ngoc Anh Thi Dang, Dan W Meyrowitsch, Ditte S Linde, Christina A Vinter, Thanh Duc Nguyen, Dang Kien Nguyen, Thi Ai Nguyen, Hieu Minh Le, Thi Kim Dung Vu, Thi Huong Tran, Tine M Gammeltoft, Jens Søndergaard

**Affiliations:** 1 Department of Environmental Health, Faculty of Public Health, Thai Binh University of Medicine and Pharmacy, 373 Ly Bon Street, Tran Lam Ward, Hung Yen Province, 17000, Vietnam, 84 0363696014; 2 Department of Public Health, Global Health Section, University of Copenhagen, Copenhagen K, Denmark; 3 Department of Gynaecology and Obstetrics, Odense University Hospital, Odense, Denmark; 4 Research Unit for Gynaecology and Obstetrics, Department of Clinical Research, University of Southern Denmark, Odense, Denmark; 5 Centre for Global Health, Department of Clinical Research, University of Southern Denmark, Odense, Denmark; 6 Steno Diabetes Center, Odense University Hospital, Odense, Denmark; 7 Faculty of Public Health, Thai Binh University of Medicine and Pharmacy, Hung Yen Province, Vietnam; 8 Department of Gynaecology and Obstetrics, Thai Binh University of Medicine and Pharmacy, Hung Yen Province, Vietnam; 9 Department of Internal Medicine, Thai Binh University of Medicine and Pharmacy, Hung Yen Province, Vietnam; 10 Department of International Relations, Thai Binh University of Medicine and Pharmacy, Hung Yen Province, Vietnam; 11 Department of Anthropology, University of Copenhagen, Copenhagen K, Denmark; 12 Research Unit of General Practice, Department of Public Health, University of Southern Denmark, Odense, Denmark

**Keywords:** gestational diabetes mellitus, self-care, digital intervention, cocreation, peer support, intervention development, health promotion

## Abstract

**Background:**

Gestational diabetes mellitus (GDM) is associated with fetal overgrowth, increased risks of perinatal morbidity and mortality, and long-term complications for mother and child, including cardiovascular disease and type 2 diabetes. Innovative, peer-based digital health interventions are emerging globally as a potential approach to assist and empower women in effective self-care and well-being during pregnancy. However, there remains substantial potential to develop and evaluate culturally sensitive digital health interventions for pregnant women with GDM, especially in low- and middle-income countries.

**Objective:**

This study aimed to bridge the gap between the World Health Organization (WHO) self-care framework and local practice by designing and piloting the “Healthy Pregnancy” intervention, a multiplatform digital ecosystem for women with GDM in northern Vietnam, through a staged cocreation and pilot refinement process.

**Methods:**

Between December 2022 and February 2024, drawing on the WHO’s conceptual framework for self-care and cocreation approach, we iteratively developed “Healthy Pregnancy,” a digital health intervention, in 4 stages: (1) formative studies and self-care construct prioritization, (2) cocreation processes with key stakeholders, (3) development and design translation, and (4) pilot testing and final refinement.

**Results:**

In stage 1, we identified gaps in current digital health interventions in low- and middle-income countries, explored the sociocultural realities of women with GDM in Vietnam, and prioritized 7 self-care constructs. In stage 2, we conducted a cocreation workshop to enable key stakeholders to co-design the foundational infrastructure for the potential intervention. In stage 3, we established a multicomponent digital intervention ecosystem with explicitly defined operating workflows. Finally, in stage 4, we gathered suggestions for a digital health intervention from a pilot test group of pregnant women with GDM, to refine and optimize the system schematic and information flow before moving to the real intervention.

**Conclusions:**

By applying a cocreation approach across all stages of development of the “Healthy Pregnancy” digital health intervention, from problem identification to solution development and evaluation, we developed a locally tailored GDM self-care model. This process not only addressed gaps in standard care but also empowered pregnant women through a supportive, multiple-stakeholder environment. This study demonstrates a rigorous cocreation pathway for systematically translating WHO self-care constructs into a feasible, culturally adapted digital ecosystem for GDM in Vietnam and offers a transferable, people-centered design process alongside a practical blueprint for integration into routine maternal care.

## Introduction

Gestational diabetes mellitus (GDM) is a metabolic health condition that affects approximately 14% of pregnancies worldwide [1]. Characterized by glucose intolerance that first appears during pregnancy, GDM, if inadequately managed, can lead to significant short- and long-term morbidity and mortality for both the mother and baby. Notably, women with a history of GDM face a 10-fold higher risk of developing type 2 diabetes (T2D) [2]. Meanwhile, their offspring not only have a higher risk of fetal overgrowth or macrosomia, one of the primary short-term complications of GDM [3], but are also more likely to have overweight or obesity [4] and have a higher risk of developing childhood- or youth-onset diabetes [5]. Lifestyle changes, including dietary modifications, a shift to an active lifestyle, and self-monitoring of blood glucose, along with medication as needed, are recommended to mitigate adverse health outcomes [6-8]. However, the complex nature of GDM and its management often pose significant challenges for women and their families [9-12]. Although education and counseling provided by health care professionals (HCPs) to women with GDM are the key components in both diabetes and pregnancy care, there are numerous challenges in the provision of standard care, especially in low- and middle-income countries (LMICs) [13].

The prevalence of GDM in Vietnam is relatively high. A study has shown that 21.8% of pregnant women in Vietnam have GDM, making Vietnam one of the countries in the Asia-Pacific region with the highest prevalence of GDM [14]. However, the health care system for pregnant women with GDM has not been sufficiently adapted to handle this situation. Due to insufficient information from HCPs and inadequate or lack of formal educational materials available [11,15], many pregnant women with GDM lack adequate knowledge about GDM and its complications and appropriate management. Additionally, cultural beliefs and traditional practices significantly affect women’s diets and physical activity during pregnancy, posing challenges to their autonomy in making informed lifestyle modification decisions [10,11].

Self-care is a well-established concept [16]; however, it has become increasingly prominent in the current era, especially since the COVID-19 pandemic [17]. To enhance the quality and equity in self-care intervention implementation, the World Health Organization (WHO) has introduced a conceptual framework for self-care within people-centered primary health care, where individuals have autonomy and are empowered to make health decisions [18]. They also require an enabling environment that includes peers, families, and HCPs to support education and counseling, ensuring equitable access to resources [18]. Therefore, in the development of self-care interventions, cocreation involving the target population, stakeholders, and researchers is essential to meet the needs of the target users and to ensure cultural and contextual appropriateness, thereby improving the acceptability and sustainability of the intervention [18-20].

Furthermore, in recent years, digital health interventions, including mobile apps, telehealth services, wearable devices, and online platforms, have emerged as promising tools for improving GDM management through timely feedback, tailored personalized care, and peer support without geographical barriers [21-24]. The latest American Diabetes Association “Standards of Care in Diabetes—2025” guideline also acknowledged that combining telehealth and in-person visits could improve health outcomes for pregnant women with GDM, compared to in-person care alone [2]. However, research on cocreating and developing such digital health interventions remains limited in Vietnam.

This study aimed to design and pilot the “Healthy Pregnancy” multiplatform digital intervention for women with GDM in northern Vietnam, explicitly mapping WHO self-care constructs to cocreated functional components and documenting the staged development and refinement process.

## Methods

### Study Design and Theoretical Framework

**Figure 1.**
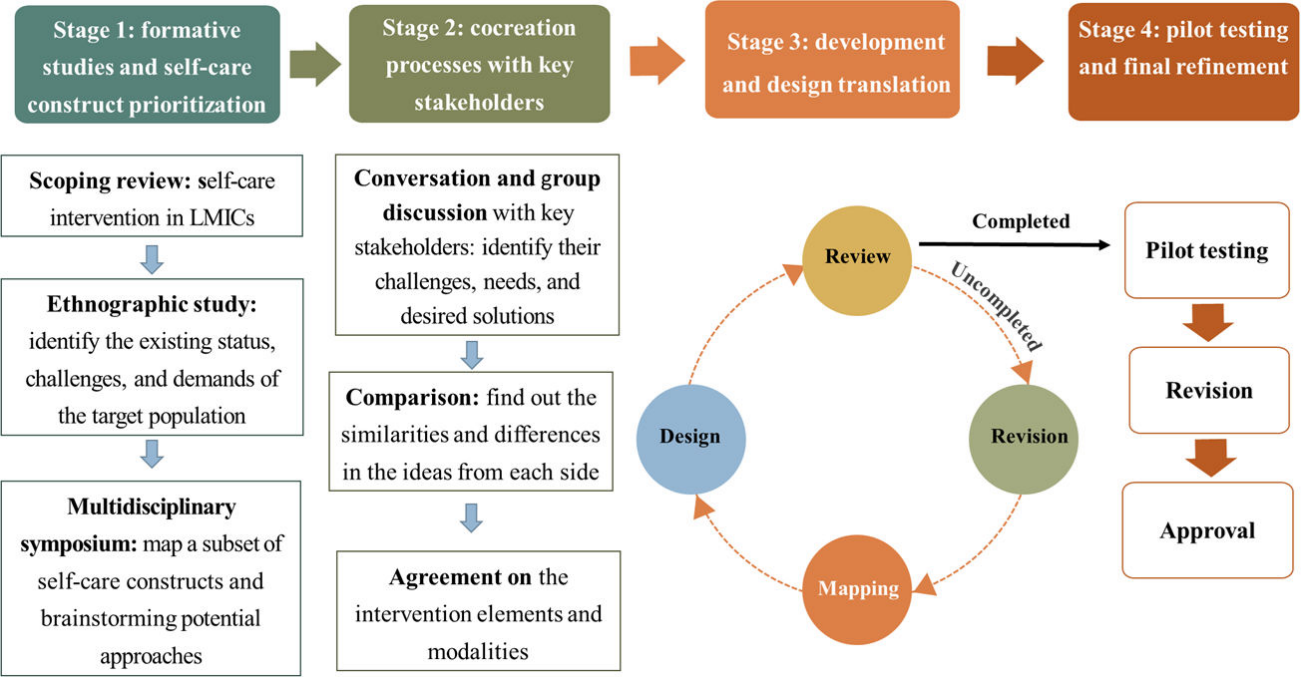
Four-stage development process of the “Healthy Pregnancy” intervention for gestational diabetes mellitus in Thái Bình (a part of Hưng Yên Province), Vietnam (May 2023-July 2024). LMICs, low- and middle-income countries.

We used a participatory cocreation design with a structure comprising 4 distinct but interconnected stages to create a new intervention initiative that meets the practical needs of pregnant women with GDM and to identify feasible design principles for a digital health intervention model suitable for the Vietnamese health care system (Figure 1).

A combination of 2 complementary theoretical frameworks was adopted—the people-centered care philosophy and cocreation design. The people-centered care approach emphasizes people-centeredness, which the WHO recognizes as a core component in the conceptual framework of self-care interventions, enabling a holistic approach that considers not only personal care but also individuals’ contexts and desires [18]. Meanwhile, the cocreation design ensured that the target populations’ roles shifted from passive recipients to active agents and coproducers of the intervention components for their health [25]. This dual-framework approach enabled us to deeply understand the sociocultural context of Vietnamese pregnant women (eg, family hierarchy and dietary traditions) and their needs, which may profoundly influence their daily self-care practices. Furthermore, the presence of cocreation across all development processes helped avoid premature, one-way solutions from the researcher’s perspective. This combination goes beyond simply gathering initiatives to foster shared priorities in self-care structures and in the desires of women with gestational diabetes, health care professionals, and research groups for digital features.

Additionally, this study was rooted in the WHO guidance on self-care interventions (2022), which expands self-care to a multidimensional set of daily practices encompassing 16 detailed, interrelated constructs divided into 3 main groups: self-management, self-testing, and self-awareness. To avoid premature selection from the researcher’s perspective, we used this multidimensional set as a comprehensive reference to inform subsequent formative studies, including a scoping review and an ethnographic inquiry, and to identify diverse stakeholders who prioritize participation in the intervention development process. Ethnography was selected to capture the sociocultural determinants of GDM self-care, for example, patrilineal decision-making, shared meal practices, and domestic power hierarchies that shape women’s daily behaviors beyond clinical settings. Data comprised repeated in-home interviews, contextual observations, photographs of artifacts, and field notes, enabling triangulation. Verbatim transcripts were coded using a thematic framework iteratively refined by the multidisciplinary team; discrepancies were resolved through consensus adjudication.

To ensure comprehensive and transparent reporting, this study followed the guidance for reporting intervention development studies in health research (GUIDED) checklist [26] and the template for intervention description and replication (TIDieR) checklist [27]. Full versions of the completed GUIDED and TIDieR checklists are available in Checklist 1 and Checklist 2, respectively.

### Study Setting and Recruitment

#### Study Setting

Thái Bình Province is in the Red River Delta Region in northern Vietnam and currently—since July 01, 2025—belongs to a part of Hưng Yên Province, according to Resolution No. 202/2025/QH15 [28]. To ensure consistency with our VALID II project [29], we have used “Thái Bình Province” in this paper. With a population of approximately 2 million, it is a rapidly developing industrial area characterized by significant socioeconomic growth. However, similar to other rapidly growing areas, Thái Bình Province is facing emerging public health challenges, particularly the rising prevalence of noncommunicable diseases such as hypertension, T2D, and GDM. In 2024, the prevalence of GDM in Thái Bình Province was reported to be 27.1% [30].

#### Standard Care for GDM

In 2022, when we designed and developed the intervention, GDM screening was not yet a mandatory step in the antenatal care process nor did the health insurance cover it [31]. In May 2024, the Ministry of Health in Vietnam launched a new guideline recommending GDM screening as a routine step in standard antenatal care [32]. Furthermore, although pregnant women can be diagnosed with GDM at either a public maternity hospital or a private clinic, they are generally advised to modify their diet by reducing white rice consumption and limiting sugar and sweets intake, as well as increasing exercise such as walking. Subsequently, they are referred to an appointment with an endocrinologist at Thái Bình Provincial General Hospital for further professional counseling on diet, exercise, self-monitoring of blood glucose, and insulin treatment, if needed, as local obstetrician-gynecologists lack competence in this specialty. If women diagnosed with GDM do not attend this appointment, they do not receive GDM self-care guidelines from the HCP [10,29].

#### Recruitment

The VALID-II project is a 2-arm nonrandomized intervention study with 2 recruitment phases. In phase 1, pregnant women with GDM were recruited for the control group. Three months later, phase 2 recruited pregnant women with GDM, who participated in the cocreated digital health intervention. We conducted recruitment at 2 health care facilities, including Thái Bình Maternity Hospital and Kim Ngan Clinic. These are 2 prestigious obstetric facilities that attract many pregnant women in the province. As a public hospital, Thái Bình Maternity Hospital is tasked with providing antenatal care and delivery services, handling approximately 11,000 births each year; meanwhile, Kim Ngan Clinic, a private health clinic, offers only antenatal care services. Detailed information on the VALID II project has been reported in a protocol paper [29].

Women contributing to the ethnographic study (stage 1), the cocreation workshop (stage 2), and pilot testing (stage 4) were recruited separately from the main intervention cohorts, wherever feasible, to minimize expectancy and contamination, with 2 prior workshop participants purposefully retained in the pilot to preserve ownership and continuity. Specific participant recruitment criteria are detailed in respective subsections describing the intervention development process.

### Development of a Content and Delivery Platform

#### Stage 1: Formative Studies and Self-Care Construct Prioritization

In the first stage, we aimed to be intentionally exploratory, informative, and nonprescriptive, which was a critical foundation for the subsequent cocreation process.

First, we aimed to gain insights into the current scientific evidence on self-care interventions for pregnant women with GDM, particularly in resource-limited areas such as LMICs. We also sought to explore culturally based self-care initiatives that address the demand for and barriers to self-care and lifestyle modifications for women and their families. Consequently, these insights informed us about the potential, up-to-date health technologies; advantages and disadvantages of the intervention models; and the existing gaps in the modalities and health aspects that each model addressed. To achieve this, we conducted a scoping review to summarize the content of self-care interventions for pregnant women with GDM in LMICs [33].

Subsequently, from a larger cohort of 233 pregnant women diagnosed with GDM in phase 1, who served as the control group in a 2-arm nonrandomized feasibility study, we randomly recruited 21 women [10,11,34-36]. These eligible women were followed up using a longitudinal ethnographic approach involving three iterative interview rounds at critical timepoints: (1) postdiagnosis, (2) postdelivery, and (3) 3 to 6 months postpartum. All participants were interviewed by the same researcher across 3 rounds, which fostered familiarity between the women and the researcher. By recruiting local interviewers who shared the same language and cultural background, we were able to accurately interpret local terminology and approach the interview with an empathetic lens, thereby avoiding personal biases and judgments and ensuring the trustworthiness of the data.

Most interviews and contextual observations were conducted within the participants’ home environments, except for the postdelivery time point, for which the interviews and contextual observations took place at the maternity hospital. The interviewers began all conversations with a slow opening and attempted to follow the flow of the participant’s narratives to create a comfortable and willing environment for sharing lived experiences through an open-ended interview guide (Multimedia Appendix 1). We used both voice recordings and detailed field notes with the participant’s consent to capture not only verbal responses but also nonverbal cues, such as gaze, gestures, cheerful ease, and hesitation and avoidance during interactions. To enrich self-reported data with actual behavior, we documented physical artifacts (eg, maternal milk supplements, dietary supplements, snacks, and self-monitoring of blood glucose) via photography. The voice recordings were transcribed verbatim by a trained transcription team and verified by the interviewer. Subsequently, a 4-member research team collaboratively developed and standardized a thematic coding framework before analyzing the data. Additionally, to address disagreements during the coding process, group meetings were held frequently under the arbitration of a senior researcher.

Finally, we organized a symposium in our multidisciplinary research team (VALID II team) to discuss, synthesize, and validate findings from the scoping review and ethnographic study, which informed stakeholder dialogues during the next stage of cocreation. This symposium included Vietnamese and Danish experts and researchers experienced in conducting self-care interventions from 3 universities: Thai Binh University of Medicine and Pharmacy (TBUMP), University of Copenhagen (UCPH), and University of Southern Denmark (SDU). The symposium participants came from various fields, including obstetrics, endocrinology, general practitioners, epidemiology, public health, nutrition, and ethnography. In this symposium, given the broad range of self-care expressions and the GDM-irrelevance of some constructs mentioned in the WHO guideline, we mapped a subset of self-care constructs most closely aligned with our project’s primary objectives: empowering pregnant women to take charge of their own health. Additionally, this subset required prioritizing relevance to daily GDM management, behavioral regulation, psychosocial well-being, and contextual relevance, as determined by the compiled findings. Furthermore, the discussions clarified key intervention methods, staffing needs, and educational materials and—drawing on VALID’s first-phase experience—identified Zalo as a promising communication platform.

#### Stage 2: Cocreation Processes With Key Stakeholders

Stage 2 aimed to translate insights into cultural GDM management, a subset of prioritized self-care pillars, and preliminary ideas from stage 1 into user-centered functional component requirements. We used a cocreation approach to foster a collaborative environment, sense-making, and negotiation, in which end users and key stakeholders acted as co-designers to weigh, prioritize, and suggest the most relevant constructs as materials for the intervention model. This stage emphasized identifying the most critical and appropriate self-care constructs, integrating them into intervention components, and considering contextual barriers before prototyping. Building on co-designs involving diverse stakeholders, these findings enabled us to balance the scientific aspects of GDM management with participants’ lived experiences.

To adopt a multidisciplinary perspective, the May 2023 cocreation workshop invited diverse stakeholders, including 8 pregnant women with GDM and their families (typically their husbands), 2 obstetricians, 2 endocrinologists, and 3 primary HCPs, facilitated by a 7-member researcher team (co-leads and notetakers). During the workshop, the facilitation team sought to reduce power imbalances between HCPs and women by centering women’s voices and strengthening their agency while still safeguarding clinical accuracy and alignment with health care system workflows.

The workshop followed a standardized script: a 15-minute opening brief; 120-minute individual, sequential conversations to surface needs, documented on flip-over charts; discussions in 4 small mixed groups to map constructs to solutions; and a 30-minute plenary session to compare the ideas and suggestions and conduct a hand vote on feasibility. Each subactivity captured and translated inputs into functional requirements. The co-leads facilitated the workshop, and the notetakers documented and photographed artifacts for analysis; the details of the workshop are summarized in Table 1 and Multimedia Appendix 2.

**Table 1.** Stakeholder-driven translation of WHO^a^ self-care constructs into digital solutions: cocreation workshop, Thái Bình, Vietnam (May 2023).

Unmet needs identified during cocreation	WHO self-care construct	Cocreated solutions	Potential platform(s) and materials
Fragmented, inconsistent, and informal GDM^b^ information	Self-education	Short, visual, practice-oriented educational materials; online counseling	Zoom (live sessions); leaflets, videos
Low confidence in lifestyle modifications; worry about the complications; feeling of loneliness in GDM management	Self-efficacy	Peer modeling through experience sharing; reassurance messages; expert-led interaction education sessions	Private Facebook page (peer posts); Zalo (peer support, messages); Zoom (live sessions)
Numerous scattered women with GDM and poor interspecialty coordination	Self-monitoring	Establishment of interactive groups by gestational week to provide timely feedback	Zalo (peer support, messages); Zoom (live sessions)
Women struggled to adjust their behaviors in real time, especially in shared family meals	Self-regulation; self-administration	Short, visual, practice-oriented educational materials for women and family members; timely nudges and reminders aligned with daily routines (eg, before meals)	Leaflets, videos, Zalo (scheduled messages, peer-to-peer image sharing)
Women lacked a safe space to share emotions, fears, and informal coping strategies without judgment.	Self-help	Moderated peer support spaces emphasizing emotional validation and shared coping	Private Facebook page (anonymous posting); Zalo groups
Need to improve autonomy in making food choices in social situations (eg, eating out and family events) or for severe cases	Self-determination	Short, visual, practice-oriented educational materials; integrating CGM^c^ technology enhances decision-making by enabling analysis of individual data.	Leaflets, videos, Zalo (guided messages, peer-to-peer image sharing); CGM devices

a WHO: World Health Organization.

b GDM: gestational diabetes mellitus.

c CGM: continuous glucose monitoring.

#### Stage 3: Development and Design Translation

On the basis of the inputs gathered from stage 1 and cocreation workshops, we aimed to translate all user requirements into a cohesive digital health–based peer support system titled “Healthy Pregnancy,” which supplements standard care for women with GDM. To ensure high accessibility and scalability in Vietnam’s cultural context and amid the digital divide, we leveraged an existing multiplatform ecosystem, including Zalo (VNG Corporation), Zoom (Zoom Communications Inc), and Facebook (Meta Platforms Inc), rather than investing in developing and prototyping a new mobile app.

The intervention suite was structured to address the 7 WHO self-care pillars concurrently. Zalo, a widely used mobile app in Vietnam, was configured as the primary tool for covering self-awareness, self-monitoring, and self-testing through a set of educational materials, peer discussions, and HCP guides. Zoom was used to host biweekly synchronous sessions for self-education, while a private (restricted-access) Facebook group provided a platform for self-awareness.

To address data privacy concerns within third-party environments, we implemented a dual-layer security strategy. First, we instructed and recommended participants to use pseudonyms and avatars instead of real photographs for all direct communication on Zalo and Zoom. Second, the private Facebook group used the “Post anonymously” feature to enable sensitive discussions without revealing identities. Moderators enforced role-based permissions, screened posts, and triaged complex queries to HCPs. Extracted data were anonymized and stored on password-protected TBUMP servers.

Regarding educational materials, we planned to provide all pregnant women with GDM in the intervention with leaflets and videos focused on topics such as diet, exercise, glucose monitoring, emotional well-being, and family support. To prepare a set of educational materials, we mapped and drafted the materials, which then underwent review rounds with experts at TBUMP, UCPH, and SDU, including obstetricians, endocrinologists, nutritionists, and health communication experts, who provided feedback and revisions on both language and visuals.

#### Stage 4: Pilot Testing and Final Refinement

In stage 4, a purposive sample of 8 women—4 pregnant women with GDM and 4 women with a history of GDM—was recruited to test the “live” ecosystem. Two participants were reinvited from the stage 2 cocreation workshop. The 6 additional participants were recruited via direct invitations at the Kim Ngan Clinic and the TBUMP endocrinology clinic, using the same demographic inclusion criteria as those used for participation in stage 2. This sampling strategy ensured that the objective would be achieved and minimized bias while preserving continuity and ownership established in the cocreation workshop.

A 2-week pilot study was conducted using a mixed methods approach to evaluate the feasibility, acceptability, and potential barriers of the multiplatform intervention before real-world implementation, without assessing clinical effectiveness.

For the quantitative assessment of educational materials, participants evaluated the readability and visual presentation of the leaflets and videos using an anonymous evaluation form (Multimedia Appendix 3). The first 4 questions referred to their impressions and assessments of the GDM leaflets or videos, covering clarity of language, font size, visual appeal, and usefulness of the content, rated on a 10-point Likert scale (1-10). Descriptive statistics were used to analyze the results. The last question was open-ended, encouraging the participants to provide additional comments or suggestions to improve the quality of the leaflets and videos.

**Figure 2.**
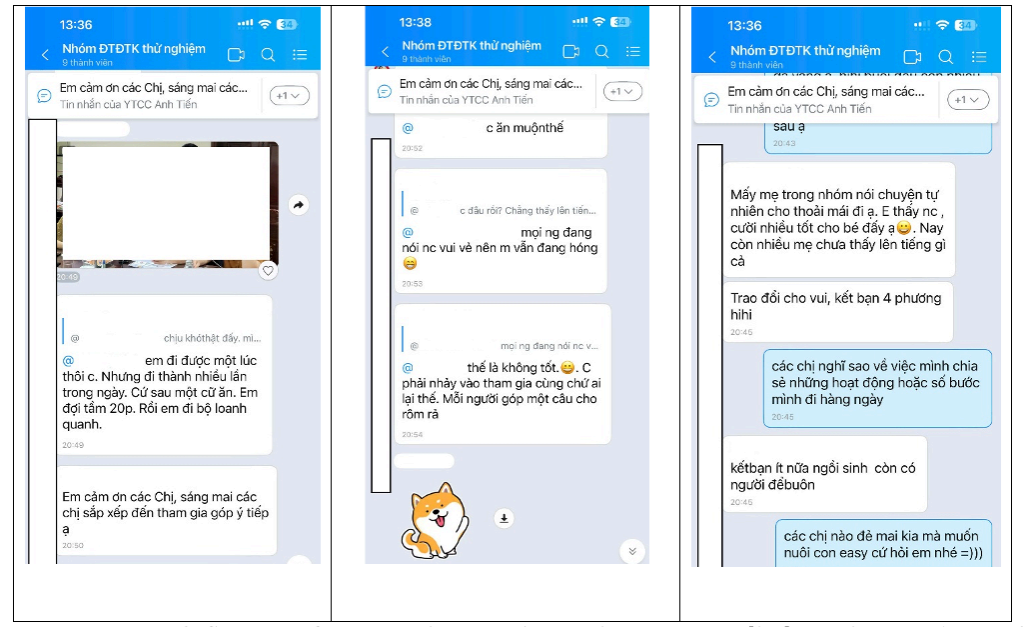
Samples of messages in the Zalo pilot testing group, Thái Bình, Vietnam, August 2023.

For qualitative observation of intervention workflow, participants were invited to join the pilot Zalo group (Figure 2), Zoom meetings, and the Facebook page for a week. The research team provided technical support to ensure continuous connectivity and identify platform-specific issues. During the pilot week, we reported patterns of interaction across platforms. Observations and implementation reflections were documented, focusing on user engagement dynamics, user preferences for interaction styles, and operational facilitators and barriers encountered in real-world use, rather than on statistics using metrics. Following the pilot phase, the 8 women met again to discuss their experiences, comments, and suggestions. Data were collected with an open-ended question guide, the moderator’s notes, and video recordings, followed by an analysis process that included structured scoring evaluation and a thematic approach. Specifically, 2 researchers independently reviewed and graded the engagement atmosphere and the quality of interactions (eg, reaction frequency and sentiment emojis). Additionally, an inductive approach was used to capture all topics and interaction preferences, while deductive codes addressed user engagement dynamics and technical barriers. Subsequently, 2 researchers cross-referenced their individual scores and notes. Any discrepancies were resolved through team consensus meetings. These insights provided valuable inputs for the final refinement before starting the actual intervention. While the pilot phase prioritized qualitative feasibility insights, the upcoming intervention phase will prospectively track platform metrics (eg, activation rates, session attendance, content views, message volumes, moderator escalations, and resolution times) to quantify acceptability and usage.

By the end of this stage, we had finalized the intervention description and submitted the revised educational materials to the Department of Information and Communications of Thái Bình Province.

### Ethical Considerations

Ethical approval for this study was granted by the Ethics Council for Biomedical Research at Thai Binh University of Medicine and Pharmacy, Vietnam, on December 1, 2022 (Institutional Review Board VN01.009). The study was registered at ClinicalTrials.gov (NCT05744856). All participants agreed and signed the written informed consent after being fully informed about the study’s objectives and procedures, including their right to withdraw at any time. All information was stored with strict confidentiality to protect participants’ identities. For the ethnographic study, the participants’ names were changed to ensure anonymity. Participants received a compensation of VND 500,000 (US $18.98) for their time and transportation costs in stages 2 and 4 and VND 200,000 (US $7.59) for each interview round of the ethnographic study.

## Results

### Stage 1: Formative Studies and Self-Care Construct Prioritization

Stage 1 provided formative insights that informed the empirical and theoretical foundation for the subsequent cocreation and intervention development stages. On the basis of the scoping review, ethnographic study, and multistakeholder symposium, we identified gaps in current digital health interventions in LMICs, explored the sociocultural realities of women with GDM in Vietnam, and prioritized 7 self-care constructs.

A total of 29 self-care intervention programs for women with GDM in LMICs, across the physical, mental, and social health dimensions, were included. Most interventions focused predominantly on improving clinical outcomes while less frequently addressing the mental and social health dimensions. Moreover, digital interventions have emerged as a promising approach to improve pregnant women’s adherence and pregnancy outcomes [33]. Additionally, interventions were often implemented and followed up only during pregnancy, overlooking the sensitive postpartum transition period.

However, although the scoping review identified a broad range of self-care constructs implemented in LMICs, no intervention studies from Vietnam were identified [32]. This underscores the need for ethnographic studies to explore the perceptions, experiences, and challenges of Vietnamese pregnant women regarding GDM self-care within the local context. Because kinship is patrilineal and patrilocal in Vietnam, self-care practices among pregnant women with GDM are deeply embedded in family dynamics and cultural dietary habits, especially influenced by the husband and mother-in-law. Decisions about dietary changes and lifestyle modifications are often profoundly influenced by the husband and, sometimes, even by the mother-in-law. Going beyond qualitative descriptive interviews that might capture only what participants report thinking and doing, the ethnographic study allowed us to explore the contextual factors, including social relations, domestic power hierarchies, and cultural norms, that shape women’s perspectives, feelings, and behaviors in daily life outside the clinical setting. Additionally, the visual records allowed us to partially assess family circumstances and identify gaps between clinical advice and real-world consumption patterns, providing insights that are often lacking in common qualitative descriptive interviews.

The ethnographic studies highlighted 2 critical barriers in the Vietnamese context. First, pregnant women were forced to become unexpected pioneers in GDM management. The lack of professional counseling in the health care system led them to seek information online. However, they expressed uncertainty about the information being heterogeneous, informal, and not culturally tailored. Second, the “shared meal” culture could profoundly influence pregnant women’s self-regulation practices because decisions of the family-in-law dictate meal preparation. Conversely, if husbands understood the psychological and practical challenges of GDM management, they could, in turn, sympathize and offer emotional support to reduce women’s overwhelming feelings. The husband’s presence could shift GDM management from a sole burden on women to a family-wide health concern, enabling a home environment that sustains self-care behaviors and improves maternal and fetal outcomes. Furthermore, through life narratives, we found that informal peer-to-peer exchanges were already occurring and were potentially valuable in some cases. In addition, the ethnographic inquiry sought to identify pregnant women’s information regarding GDM, their preferences for specialist advice, and potential effective self-care constructs for pregnant women with GDM that they discovered through personal experience. These insights justified the need for the people-centered care framework to address the “whole person” within a woman’s family and social context, beyond clinical indices.

Based on the formative studies, we synthesized and validated the inputs for presentation at the symposium. To translate these broad insights into a structured intervention, the WHO’s 3 main pillars and 16 self-care constructs were systematically assessed at the expert symposium, with an emphasis on clinical management, well-being, and contextual relevance for the Vietnamese population with GDM. We identified and mapped several problem-need clusters relevant to women with GDM onto multiple WHO self-care constructs. This reflected the interconnected, synergistically reinforcing nature of self-care constructs in GDM management rather than their functioning independently. Additionally, empowering women in home-based GDM self-management was the primary purpose rather than clinical diagnostics or treatment delivery, leading to the exclusion of constructs reliant on these. Therefore, we prioritized 7 WHO self-care constructs as most actionable for home-based GDM management, including self-education, self-efficacy, self-monitoring, self-regulation, self-help, self-determination, and (context-specific) self-administration, on the basis of clinical relevance, feasibility, and stakeholder consensus (Table 2).

**Table 2.** Mapping scoping and ethnographic findings to WHO^a^ self-care constructs for GDM^b^: cocreation study, Thái Bình, Vietnam (2023).

Relevance to women with GDM (key findings) and WHO self-care construct (2022)	Resources	Prioritization decision	Reason for prioritization (link to GDM management and well-being)
Key gaps and unmet needs			
Need for holistic care: integrating mental and social health into GDM management			
Self-efficacy Self-education	Scoping review [33]	High	Self-efficacy in self-care capacity may influence mental health and maintain long-term behavioral changes. Additionally, GDM awareness reduces anxiety and improves glycemic control.
A longer follow-up and support			
Self-education Self-regulation Self-monitoring Self-help	Scoping review [33]	High	Longitudinal support fosters sustainable, peer-based resilience; reinforces health literacy; and ensures consistent self-monitoring postpartum, facilitating proactive behavioral regulation.
Lack of GDM awareness and confusion among the abundance of information on the internet			
Self-education	Ethnographic studies [10,11,36]	High	Requires official, standardized medical information that supports pregnant women with an accurate understanding
Barriers to shared meal culture and lack of family support			
Self-help Self-administration	Ethnographic studies [10,11,36]	High	Engaging family support to overcome “shared meal” challenges and implement daily modifications
Desire for an effective support environment			
On-time support; elimination of geographical or financial barriers			
Self-education Self-regulation Self-monitoring	Scoping review [33] Ethnographic studies [35]	High	GDM requires daily modifications. Immediate feedback or guidance empowers women to promptly understand and self-regulate their lifestyle and maintain self-monitoring of blood glucose.
Safe and no-blame space for exchanging experiences and emotions			
Self-help Self-determination Self-efficacy	Symposium	High	A safe, accompanied peer support environment fosters self-determination and self-help and boosts mental health, self-confidence, and social connection.
Requirement of structured and visual educational materials (videos/leaflets)			
Self-education Self-monitoring	Ethnographic studies [35] Symposium	High	Providing accurate medical information via visual aids helps women acquire new knowledge more easily and is suitable for all educational levels.
Other clinical suggestions			
Clinical diagnostics: sampling, screening, and collection of medical samples			
Self-sampling Self-screening Self-diagnosis Self-collection	Symposium	Excluded Excluded Excluded Excluded	These procedures are typically performed in hospitals under medical supervision.
Medical treatment			
Self-injection	Symposium	Low	In Vietnam, pregnant women with GDM use only insulin.
Self-medication Self-treatment Self-examination Self-use	Symposium	Excluded Excluded Excluded Excluded	Interventions focused on empowering women to build WHO self-awareness constructs, thereby promoting lifestyle modification at home rather than complex medical procedures.

a WHO: World Health Organization.

b GDM: gestational diabetes mellitus.

### Stage 2: Cocreation Processes With Key Stakeholders

Stage 2 aimed to translate stage 1 findings into user-centered functional requirements and components, thereby establishing the intervention’s foundational infrastructure, through 3 main activities with valuable insights from diverse key stakeholders during the cocreation workshop.

#### Gaps in Professional Interaction and GDM Care Within Local and National Health Care Systems

In the initial activities, a realistic picture of fragmentation in GDM care within the health care system was reported, reconfirming the stage 1 findings. Three core issues were (1) a lack of standardized official information and counseling channels for women with GDM and their families; (2) limited endocrinologist-obstetrician interaction during follow-ups and childbirth; and (3) high expectations for physician comprehensiveness (Multimedia Appendix 2).

An obstetrician admitted the challenges in counseling:

The obstetricians had limited specialized knowledge of endocrinology and GDM…Pregnant women expect doctors to have comprehensive training in all diseases, which makes it challenging to provide satisfactory advice.

Endocrinologists reported challenges from inadequate equipment, limited awareness of GDM among pregnant women and the community, lack of official local information channels, and limited interaction with obstetricians during follow-ups for pregnancy and childbirth.

This status led to frustration with unmet needs in professional care and confusion about the overwhelming heterogeneity of online information. An expectant mother, aged 40 years, participating in the workshop shared the following:

I wanted to know what kind of diet is for people with GDM; I did my own research on the Internet and found that it is very dangerous for both mother and child… I adjusted my diet, but sometimes I had low blood sugar. My diet was not scientific, but I did not find a reasonable solution.

#### Prioritize and Translate a Subset of WHO’s Self-Care Constructs Into Potential Solutions Grounded in Realistic Stakeholder Insights

In the group discussion activity, participants mapped unmet needs and gaps to 7 prioritized WHO self-care constructs, translating them into concrete functional requirements and selecting feasible delivery platforms (Table 1).

For example, the lack of formal information channels or confusion from unverified internet sources, which fit the self-education construct, led to a demand for an expert-led system of video tutorials and leaflets. To bridge the gap in endocrine expertise among obstetricians and the limited interaction between obstetricians and endocrinologists, online counseling groups facilitated women’s self-monitoring and self-regulation, particularly those in scattered populations. Similarly, other critical challenges for women included a lack of confidence and feelings of loneliness during GDM management, which requires self-efficacy and self-help. In response, the stakeholders emphasized the importance of peer modeling, leading to the design of peer support groups for sharing rather than solely expert-led education.

Additionally, rather than orienting the participants to predefined technological solutions, we encourage them to self-propose ones based on accessibility, familiarity, and privacy. The integrated, multiplatform approach, which included existing platforms such as the Zalo mobile app, Zoom video conferencing, and Facebook, was identified as a suitable infrastructure that addressed geographical barriers and empowered pregnant women within the context of family culture in Vietnam. Every requirement regarding the organization and operational format of the platforms was documented on flip-over charts by each group and incorporated into the subsequent discussion and deliberation process (Multimedia Appendix 2).

After the groups presented their ideas, we shared, compared, and reached consensus on potential solutions for implementation in the trial and for future scale-up. We reached consensus on establishing small Zalo groups, Zoom online counseling, family groups, private Facebook groups, and visual educational materials. We also agreed on the need to improve the capacity of HCPs in standard care for GDM through short training courses. However, the idea of continuous glucose monitoring was eliminated due to its high cost, which was unsuitable for all women with GDM (see Table S1 in Multimedia Appendix 2).

Consensus outputs (Table 1) were converted into technical specifications and scripts that informed the platform configurations summarized in Table 3, including triage workflows and standardized nudge libraries.

**Table 3.** Technical configuration and behavioral mechanism of the “Healthy Pregnancy” ecosystem for GDM:^a^ Thái Bình, Vietnam (2023‐2024).

Platform component	Technical configuration and workflow	Behavioral mechanism	Final design artifacts
Zalo: interaction tool	Gestational week–based peer support groups implementing a triage workflow: initial peer support addresses simple inquiries; if complex issues: moderator receiving information → escalating to HCPs^b^ in group and specialists in Zoom meetings	Immediate feedback and cues to action enable rapid behavioral adjustment and minimize confusion and anxiety.	Welcoming, introduction, and nudge message scripts; moderator dispatch procedures
Zoom meetings: online counseling	Scheduled online counseling sessions with specialists in obstetrics, endocrinology, and nutrition	Enhancing GDM awareness and reinforcing self-efficacy through guided problem-solving	Preschedule online specialist consultations and prepare corresponding discussion topics for each.
Private Facebook page: resource center	Structured content library aligned with pregnancy progression; integrated anonymous posting feature	Enabling women to self-educate, receive peers’ empathy, and share without judgment	Official, standardized educational materials (leaflets and expert-led videos)

a GDM: gestational diabetes mellitus.

b HCP: health care professional.

### Stage 3: Development and Design Translation

At this stage, the ideas generated during the cocreation workshop were translated into a multicomponent digital intervention ecosystem with explicitly defined operating workflows. Rather than being designed and implemented as a standalone, completely new mobile app, the intervention integrated educational, behavioral, and psychosocial support via familiar digital platforms to empower women’s daily self-care practices. These mechanisms were designed to operate concurrently and complementarily throughout the intervention period, addressing interrelated aspects of self-care capacity (Table 3).

To ensure an effective and engaging intervention, we developed concise, culturally relevant, and accessible educational materials across 3 aspects of GDM self-care. The set of educational materials included 8 leaflets (1 print and 7 digital) and 8 videos (see Multimedia Appendix 4). The visual elements used simple language, illustrations, and real-life scenarios to enhance engagement and facilitate the daily application of self-care principles by pregnant women and their families. Diverse experts, including obstetricians, endocrinologists, public health professionals, and health communication specialists, reviewed and provided feedback on all educational materials. This process ensured the refined files’ medical accuracy, feasibility, and effective message delivery through the use of simplified language and enhanced illustrations and improved cultural relevance.

To fulfill its commitment to supportive spaces and protect participants’ rights, the “Healthy Pregnancy” intervention established security measures across all platforms. In Zalo groups, we implemented hierarchical data management, with moderators responsible for screening members, securing discussion content, and limiting information sharing to the framework of professional treatment consultations with HCPs. Additionally, women were instructed to use pseudonyms and avatars instead of real photographs for all communication via Zalo and Zoom meetings. The Facebook page was set to private mode, allowing access to only approved, project-verified members, to prevent leaks. Participants on the Facebook page also used the “Anonymous Posting” feature to share about psychological pressures from family or experiences without fear of judgment or identification.

### Stage 4: Pilot Testing and Final Refinement

#### Pilot Testing With Educational Materials

Table 4 presents the pilot participants’ responses to the educational materials in the intervention. Most of the pilot participants (87.5%, 7/8) rated the language used in the leaflets and videos as 7 to 9, indicating understandable language, while 1 participant (12.5%) rated it 6. The mean rating was 7.6 (SD 0.9). Regarding font size readability, most (75%, 6/8) participants gave a positive rating of 7 or 8 for ease of reading, while 25% (2/8) of the participants rated it 6. The visual elements were rated with an average score of 7; most found them attractive and engaging (7 or 8 points), while 1 participant rated them as 6, and another rated them as 5. Regarding the usefulness of the content, all participants assigned a high score between 7 and 10, with an average score of 8.6 (SD 0.9).

**Table 4.** Language and cultural evaluation of materials during pilot testing of a GDM^a^ intervention with 8 participants: Thái Bình, Vietnam (August 2023).

Criterion	Score^b^, mean (SD)	Score range
Language	7.6 (0.9)	6‐9
Font size	7.3 (0.9)	6‐8
Visualization	7.0 (1.1)	5‐8
Content	8.6 (0.9)	7‐10

a GDM: gestational diabetes mellitus.

b All items were rated on a Likert scale from 1 to 10, where 1 indicates “very poor” and 10 indicates “excellent.” Higher scores represent greater approval and cultural appropriateness of the materials.

Responses to the open-ended question showed that most participants appreciated our educational materials. They suggested revising the wording to increase accessibility to pregnant women, explaining medical terms, increasing font size for easier reading, and using images of pregnant women and foods that reflect Vietnamese culture.

### Pilot Testing With the Intervention Process

Observations and discussions during pilot testing indicated high engagement in Zalo groups through messages, images, and emojis. Apart from exchanging GDM self-management experiences, the women seemed to have conversations that extended to broader aspects of daily life and family contexts. Additionally, Zoom-based online consultations with HCPs were well received for addressing unanswered questions during brief clinical visits.

On the other hand, some observed operational challenges included variations in interaction dynamics depending on whether active participants were present in the Zalo group. In Zoom sessions, engagement and interest decreased when didactic clinical content dominated the sessions, compared with sessions structured as an interactive question-and-answer section. Additionally, unstable internet connectivity was reported as a technical barrier that disrupted live consultations. Anonymous posting on Facebook was not preferred, as most informational needs were addressed through Zalo and Zoom.

Consequently, we refined and standardized the implementation process. Zalo was designated as the primary platform for daily interaction, and active women were encouraged to initiate discussions. Zoom sessions were restructured into short lectures and a case-based, question-driven format with a flexible duration. Additionally, we designated Facebook as a repository for educational materials and information, with pinned posts to ensure stable, easy-to-locate access. To enhance system credibility, a formal project logo was designed on the basis of the majority preference (6 of 8 participants), and HCPs were required to use standardized identification, including full names and professional titles, across all digital groups. Finally, we implemented an operational adjustment: integrating QR codes into the implementation workflow to facilitate women’s access to information.

After refining the intervention through multiple rounds of feedback and revisions, we submitted the educational materials and received approval from the Department of Information and Communications of Thái Bình Province.

Finally, we also completed the (1) client flow from diagnosis to postpartum, (2) moderation and triage logic, and (3) examples of scripts, applying to the intervention for pregnant women with GDM (see Multimedia Appendix 5).

## Discussion

### Principal Findings

This study provides empirical evidence for the systematic development and pilot refinement of the “Healthy Pregnancy” digital peer support ecosystem, demonstrating how it operationalizes global self-care frameworks within local clinical practice in Vietnam. Through a rigorous, 14-month-long, staged process, we operationalized 7 prioritized WHO self-care constructs into an integrated multiplatform architecture, leveraging the Zalo mobile app for real-time interaction and peer support, Zoom meetings for expert-led counseling, and Facebook as a static resource hub. Unlike previous formative research from our group, this study uniquely explicates the design translation process, moving beyond “identifying needs” to document how real-world demands and WHO self-care constructs were integrated and translated into concrete digital features and behavioral mechanisms.

Furthermore, a functional peer support workflow was designed to address the fragmented coordination between obstetricians and endocrinologists. The intervention’s educational materials package, including 8 sets of practical videos and educational leaflets, was cocreated with stakeholders acting as co-designers rather than passive informants. Additionally, pilot testing confirmed the feasibility of this multiplatform approach, which revealed high engagement in Zalo groups and the need for interactive, case-based Zoom sessions. Moreover, final refinements, such as an official project logo and standardized HCP identification, were led by end users, serving as trust-building mechanisms.

### Interpretations in Context

To the best of our knowledge, this study is the first to describe the multistage development of a digital health–based peer support intervention for pregnant women with GDM in Vietnam. Uniquely, we made the often-implicit design logic explicit, mapping global self-care constructs onto concrete digital components and user journeys suitable for public maternal care in a lower-middle-income context. One of the key strengths of our study was its comprehensive, participatory, and contextualized approach, which incorporated the WHO’s conceptual framework for self-care and the cocreation method across multiple stages. Drawing on the WHO’s conceptual framework for self-care [18], we established a digital health ecosystem focused on people-centered primary health care, integrating structured educational materials, expert-led counseling, and peer support to empower pregnant women to make informed decisions and adhere to daily self-care behaviors.

Meanwhile, engaging the target users and multiple stakeholders, the cocreation approach enabled the intervention content and modalities to be adjusted relevant to the target group’s context and preferences [19,37,38]. The cocreation process in VALID-II was well demonstrated in co-leadership in prioritization and shared ownership of design choices. Participants acted as co-designers by proposing, debating, and voting on delivery modalities (eg, Zalo group size and Zoom timing) and removing infeasible ideas (eg, continuous glucose monitoring), with researchers implementing these joint decisions. To enhance system credibility, a formal project logo and standardized HCP identification were implemented across all digital groups, the basis of the majority preference. These refinements, driven by stakeholder leadership, confirm a transition from conventional qualitative inquiry to true shared ownership of the endeavor.

This systematic yet flexible procedure enabled us to apply up-to-date scientific insights effectively and to modify the intervention program locally to the context and needs of the target audience. It also aligns with the design of complex interventions, particularly in resource-limited settings, where cultural appropriateness and stakeholder engagement are key elements for effectiveness and sustainability.

Another strength of our study was the ability to leverage widely available, low-cost digital platforms, including Zalo [39], Zoom [40,41], and Facebook. Our cocreation pathway goes beyond education-only designs by linking prioritized self-care constructs (self-education, self-efficacy, self-monitoring, self-regulation, self-help, self-determination) to specific platform features and behavioral mechanisms (eg, cues to action via Zalo nudges; problem-solving efficacy via Zoom question-and-answer sessions; anonymous peer validation via Facebook). Beyond the familiarity and feasibility of Vietnam’s technological infrastructure, this ecosystem helped blur geographical barriers, facilitate timely support and counseling, and reduce pressure on the professional health care system. Moreover, the accompaniment of peer support and family within this ecosystem provides social validation and habit scaffolding, which were essential for sustaining lifestyle changes within the Vietnamese “shared meal” culture, where family dynamics often dictate dietary and exercise habits [42-45].

Finally, design choices such as leveraging widely adopted platforms, standardizing moderation standard operating procedures, and supporting low-bandwidth content help scale up and maintain fidelity within routine antenatal care, creating favorable conditions for sustaining and scaling the model in LMIC settings such as Vietnam. The intervention was intentionally designed around task shifting, with moderation and first-line triage delivered by trained midwives or diabetes nurses and escalation to obstetricians or endocrinologists only when clinically indicated, thereby minimizing additional workload while preserving patient safety. In parallel, women with GDM were engaged as peer knowledge contributors within clearly defined nonclinical boundaries, reducing repetitive informational demands on health care staff while strengthening comfort, connectedness, and mutual support. From a governance perspective, early incorporation of standardized HCP identification and structured moderation procedures embeds accountability mechanisms from the outset, providing a foundation for traceable data flows, referral documentation, and future interoperability with the Ministry of Health systems. The development of structured training pathways for moderators and specialist responders will be critical to ensure long-term sustainability.

### Key Implications

The key implications of this study are as follows:

1. A transparent mapping from global self-care constructs to local digital features is feasible.
2. Cocreated moderation and triage processes are essential to sustain engagement.
3. Ubiquitous platforms can deliver timely, low-cost support at scale.
4. Formal data governance must be designed in from the outset.

### Limitations

However, our study had several limitations. First, the digital health intervention was designed specifically for pregnant women with GDM in Thái Bình Province, northern Vietnam, leading to its highly context-specific nature. Although its design and development process could be fully transferable, replicating the model without adapting to cultural and social norms, digital access, online platforms, or the health care system may involve difficulties.

Second, although 79.1% of the population in Vietnam has internet access [46], and we tried to use freely accessible digital platforms locally, it may still not fully cover individuals without stable internet access. Furthermore, digital literacy was a significant barrier to the adoption of digital health, leading to the requirement of accompanying technical support and training [47]. Therefore, they may inadvertently limit the access of socioeconomically disadvantaged and low-digital-literacy groups to the intervention.

Third, while we expected that it would be feasible to integrate the intervention into standard maternal care, it still required an available staff team for management and moderation, as well as a specialist team for education and timely counseling. Another potential limitation of online peer group interventions is participants’ reluctance to share their thoughts and emotions with unfamiliar peers, which may hinder group cohesion and reduce the intervention’s overall effectiveness. However, these participants may still benefit from the information or experiences shared by other members and professional advice from health care workers in the group. Additionally, the design and development process took a long time, 14 months. Therefore, it may not achieve ideal cost-effectiveness in this period. Nevertheless, this process serves as an essential stepping stone, as we can leverage its advantages and results in the next phase of the VALID study.

### Conclusion

This study demonstrates a rigorous cocreation pathway that systematically translates the WHO self-care constructs into a feasible, culturally grounded, multiplatform digital ecosystem for GDM in Vietnam. By making the design logic, behavioral mechanisms, and implementation workflow explicit, we contribute a transferable method for building people-centered digital self-care interventions in resource-constrained health systems and a concrete blueprint for their integration into routine maternal care.

## Supplementary material

10.2196/82434 Multimedia Appendix 1 Open-ended interview guide.

10.2196/82434 Multimedia Appendix 2 Details of the cocreation workshop with key stakeholders.

10.2196/82434 Multimedia Appendix 3 Evaluation form for educational materials.

10.2196/82434 Multimedia Appendix 4 Overview of educational materials.

10.2196/82434 Multimedia Appendix 5 System schematic and information flow at Thai Binh University of Medicine and Pharmacy (TBUMP).

10.2196/82434 Checklist 1 The guidance for reporting intervention development studies in health research (GUIDED) checklist.

10.2196/82434 Checklist 2 The template for intervention description and replication (TIDieR) checklist.

## Abbreviations

GDM: gestational diabetes mellitus
GUIDED: guidance for reporting intervention development studies in health research checklist
HCP: health care professional
LMICs: low- and middle-income countries
SDU: University of Southern Denmark
T2D: type 2 diabetes
TBUMP: Thai Binh University of Medicine and Pharmacy
TIDieR: template for intervention description and replication checklist
UCPH: University of Copenhagen
WHO: World Health Organization
