# Supplementary material for: Digital Health–Based Peer Support Ecosystem for Gestational Diabetes Mellitus in Vietnam (VALID II Study): Multistakeholder Cocreation and Pilot Study
Source: J Med Internet Res. 2026 May 6;28:e82434. doi: 10.2196/82434 (PMC13148331; doi:10.2196/82434)
Supplement: Multimedia Appendix 1 [file jmir-v28-e82434-s001.docx]

## Multimedia Appendix 1. Open-ended interview guide.

**Interview guideline for WP2:**

**Pregnant women’s experiences and perceptions of GDM**

**Start with**: Introductions, small talk

*Throughout: Bro.ad and open questions to allow space for her perspective. Try to turn the interview into a natural conversation, with the flow of a natural conversation = go with the respondent, try to follow her flow.*

**Themes:**

**Theme 1: Family life**

**Please tell us about your pregnancy and your family.**

*Probes:*

How many children do you have? How old are they?

Who lives in your household?

Who do you get along with the best/who supports you the most?

Does your family of birth live far from here?

The pregnancy: was it planned? How did you feel when you learnt you were pregnant?

**Theme 2: The GDM diagnosis**

**Please tell us about the GDM diagnosis: what happened, and how did you feel?**

*Probes:*

When and where were you diagnosed with GDM?

How did you feel at the time of diagnosis?

Had you ever heard about GDM before?

What is GDM? Do you understand it?

If you compare your experience of pregnancy *before* the GDM diagnosis and *after*, what are the differences?

How did family members react to the diagnosis at the time of diagnosis and now? (probes: husband, parents-in-law, parents)

What is GDM, as your family members understand it?

Are any of your family members living with T2D or GDM?

**Theme 3: Pregnancy self-care in general**

**Please tell us about how you cared for yourself as a pregnant woman before the GDM diagnosis? Who supported you the most?**

*Probes:*

Who in the family (husband’s family + family of birth) is involved in your pregnancy care?

In general, what do you think is good for a mother’s health during pregnancy? (focus on mother, not child)

In general, what can damage a mother’s health during pregnancy? (focus on mother, not child)

If you have been pregnant before, please describe how you cared for yourself during previous pregnancies.

In general, what do you see as the dangers during pregnancy/what can harm a developing fetus? (probes: pollution, toxic foods, alcohol/smoking, mother’s moods, etc.)

In general, what do you think strengthens a developing fetus? (probes: diet, rest, dietary supplements, mother’s peace of mind, teaching baby in the womb, etc.)

Did you meet any obstacles/difficulties in self-care before the GDM diagnosis?

**Theme 4: Being pregnant with GDM – self-care and social support**

**Please tell us about your life as a pregnant woman with GDM. How are you, and what are your thoughts, feelings, and actions?**

**Please describe who supports you in handling the GDM during this pregnancy?**

*Probes:*

How has self-care changed since you knew about the GDM? (diet, exercise, blood sugar control)

Which fears, worries, or concerns do you have? How have your feelings changed over time since you got the diagnosis?

What challenges do you meet? (probes: diet, controlling blood sugar, etc.)

If you have been pregnant before, without GDM, what are the differences between the pregnancies in terms of how you feel?

What support do you receive from family members?

Do you feel that your family understands you and the challenges you meet? (talk about each person in the family and their reactions to the GDM)

Do you feel relations with family members (natal family, husband’s family) have changed because of the GDM? (for better or for worse?)

Please tell us about the support you have received from healthcare providers.

**Theme 5: Life just now: any burning issues apart from the GDM?**

Please tell us briefly about your life just now: apart from the GDM, are you and your family currently facing other challenges?

**Theme 6: The future**

**What are your thoughts about the future?**

*Probes:*

Pregnancy care from now on

Plans for the delivery?

Fears/concerns regarding the child’s health?

Fears/concerns regarding own health?

**Wrapping up:**

Do you have any questions for us?

Make an appointment for the next visit.
