# Supplementary material for: Digital Health–Based Peer Support Ecosystem for Gestational Diabetes Mellitus in Vietnam (VALID II Study): Multistakeholder Cocreation and Pilot Study
Source: J Med Internet Res. 2026 May 6;28:e82434. doi: 10.2196/82434 (PMC13148331; doi:10.2196/82434)
Supplement: Multimedia Appendix 2 [file jmir-v28-e82434-s002.docx]

# **Multimedia Appendix 2. Details of the cocreation workshop with key stakeholders**

## **The participants in the cocreation workshop:**

To ensure a genuinely multidisciplinary approach, the cocreation workshop held in May 2023 brought together a broad and diverse group of stakeholders, including eight pregnant women with GDM and their families (typically their husbands), two obstetricians, two endocrinologists, and three primary HCPs, facilitated by a seven-member researcher team (co-leads + note takers). The pregnant women were recruited from phase 1 or referred by obstetricians from Thái Bình Maternity Hospital or Kim Ngan Clinic, unlike those in the stage 1 ethnographic study. A team of seven Danish and Vietnamese researchers (VALID II team) with user-centered design expertise facilitated the cocreation process. Two lead facilitators guided discussions and presented preliminary initiatives, while six supportive facilitators took notes, observed, and assisted participants.

## **The cocreation process**

The workshops were consistently structured into three main sections with an opening, a core sequence of activities, and a brief conclusion. A 15-minute opening was implemented to establish context, introduce the VALID II project's purpose, the facilitator, and participants, and emphasize the co-design foundation rules to foster active participation. The core activity section lasted 120 minutes and included three sequential activities using a mixed qualitative approach, combining individual conversations and four group discussions.

In the first activity, we conducted individual conversations between the facilitator and each participant to reconfirm the demands, difficulties, and gaps in maternal care and GDM self-care within the current health system from the perspectives of key stakeholders. An observer briefly recorded each opinion and perspective on the flip-overs. Additionally, we presented the subset of WHO self-care expressions and the voting for prioritization requirements in the next activity. Following that, the participants were divided into four small-group discussions, comprising two women and their families, one obstetrician or endocrinologist, and one to two HCPs. One or two researchers participated in each group to facilitate group discussions, encourage active engagement, and take notes. They were required to map the self-care constructs according to their needs and to brainstorm specific intervention components, features, and delivery modalities tailored to the end user's daily challenges. In the last activity, we created an opening space to encourage participants, especially women and their families, to stand up and present the outcomes of the discussion group.

In the last 30 minutes of the workshop, we shared our stage 1 insights and preliminary ideas, thereby comparing and summarizing the similarities and differences between our initial ideas and the participants' contributions. To address the differences, we used verbal discussion to gather views on the advantages and disadvantages, and we voted by hand-raising on the feasibility of intervention components and delivery modalities.

## **The results of the cocreation workshop**

1. **Sub-activity 1: The unmet need and disconnection in GDM care**

| 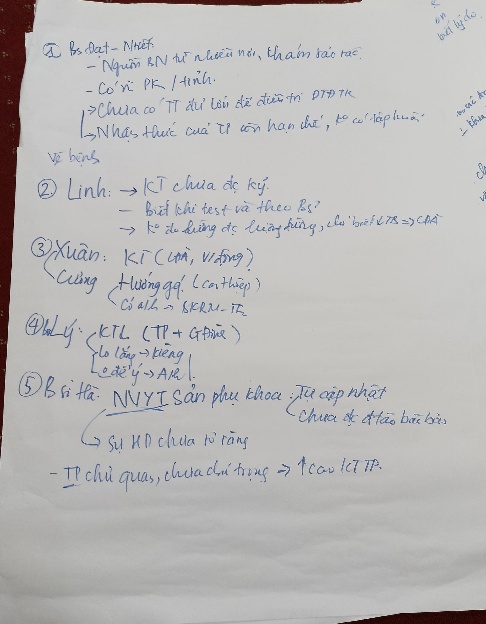 | 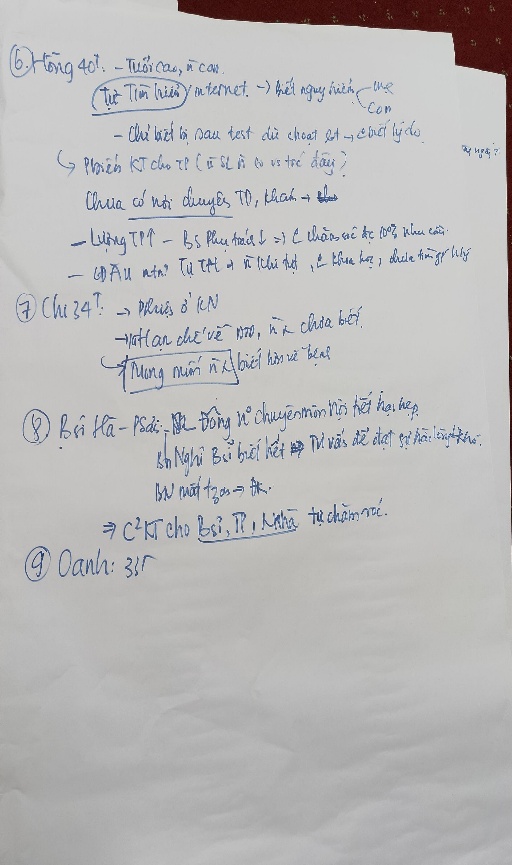 | 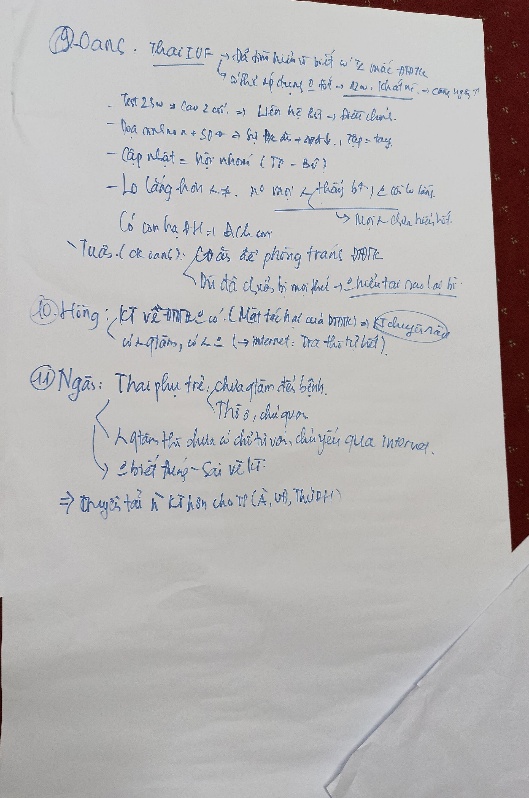 |
| --- | --- | --- |

**Figure S1-S3. Examples of the unmet need and disconnection in GDM care in cocreation workshop, Thái Bình, Vietnam (May 2023)**

1. **Sub-activity 2: Group discussion**

| 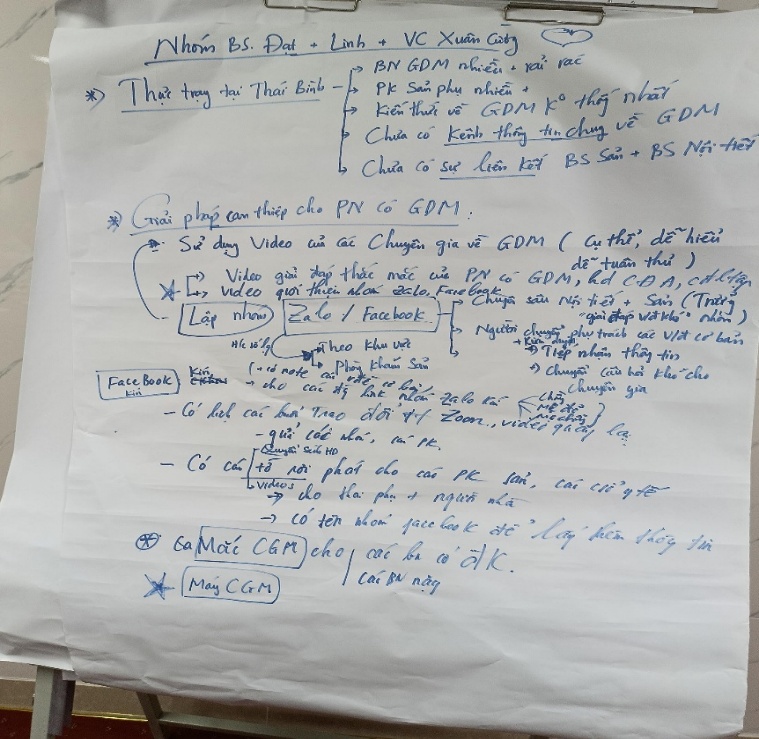 | 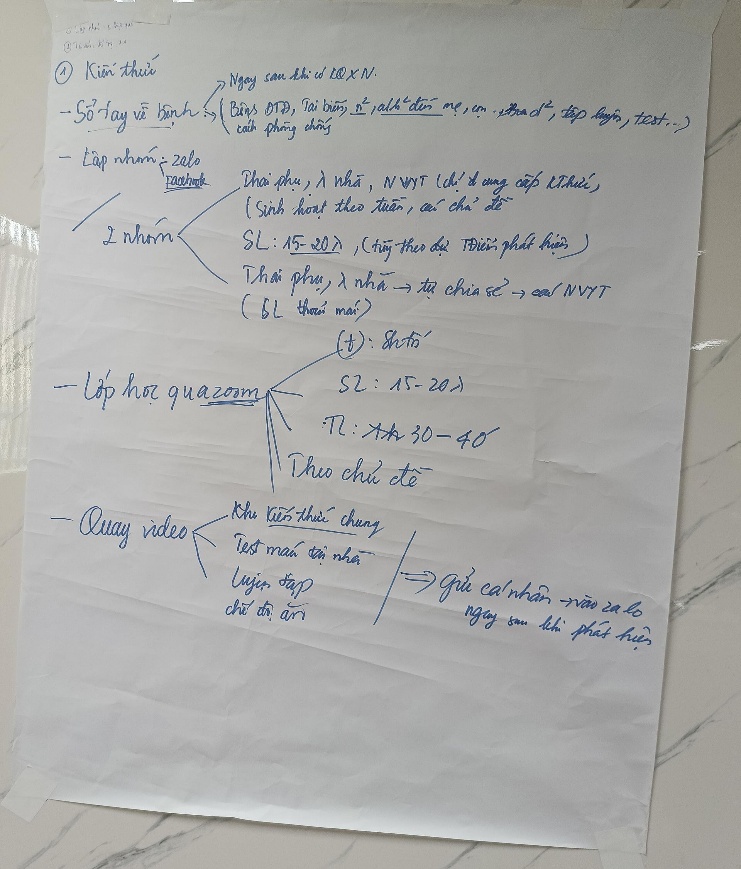 |
| --- | --- |

**Figure S4-S5. Examples of a cocreation artifact generated in group discussions, illustrating the translation of identified gaps into candidate intervention components (raw outputs).**

*The English translation of examples of a cocreation artifact generated in a group discussion*

The participants also expressed interest in each platform. For example, a small Zalo group of 15-20 members at a similar stage of their GDM diagnosis was preferred. They also mentioned the desire for advice from endocrinologists, obstetricians, and peers. For Zoom sessions, the ideal time would be 30 to 40 minutes at 8 pm on a fixed day, covering different topics. A private Facebook group was also recommended to store educational materials and activities.

Regarding educational materials, the groups discussed and agreed that both leaflets and videos should be provided, offering guidance on diet, exercise, and home blood sugar monitoring. The educational materials should be clear, easy to follow, and written in simple, accessible language. In addition, one group suggested that there should be training courses for HCPs on GDM at Thái Bình Maternity Hospital and obstetrics and gynecology clinics. Another group mentioned continuous glucose monitoring (CGM) could be considered in severe cases or for women in favorable economic situations.

### **Table S1. Consensus on intervention modalities and content: Cocreation workshop, Thái Bình, Vietnam (May 2023).**

| **Intervention ideas from group discussion** | **Intervention delivery** | **Applying (Y/N)** | **Reason for applying or not** |
| --- | --- | --- | --- |
| Small Zalo groups | Consists of 15–20 members at similar gestational ages.   - Have the support of HCPs and peers - 01 admin to manage, collect the information, and transfer the difficult questions to the specialists. | Yes | An ideal number of members.  Similar ideas as outlined in Stage 1 |
| Zoom meetings | - Lasted 30 - 40 minutes, starting at 8 pm on a fixed day - Discussion on a specific topic with specialists | Yes | Zoom meetings offer a free 40-minute version, which facilitates the trial and scaling up.  Similar ideas to those outlined in Stage 1. |
| Family group | - Invite husband or mother/mother-in-law for knowledge sharing | Yes | Similar ideas to those outlined in Stage 1. |
| Private Facebook Group | - Admins (researcher team): moderate posts, answer the questions, share Zoom links, and record videos | Yes | Similar ideas to those outlined in Stage 1. |
| Educational materials | - Leaflets - Videos | Yes | Similar ideas to those outlined in Stage 1. |
| Training workshop for HCPs | - Short courses for obstetricians and nurses | Yes | Need to improve HCP capacity in standard care for GDM. |
| Use Continuous Glucose Monitoring (CGM) | For severe cases or women in favorable economic situations. | No | CGM requires high costs and is unsuitable for the trial and scaling up. |

## **Final refinements from the end users: A formal project logo and a sample of the revised leaflets**


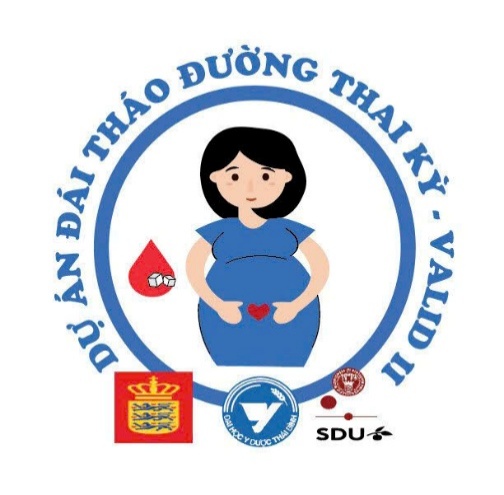


**Figure S6. The final formal logo of the "Healthy Pregnancy" digital health-based peer support intervention**

### **A sample of the revised leaflets before and after the pilot testing**

| **Before** |  | **After** |
| --- | --- | --- |
| **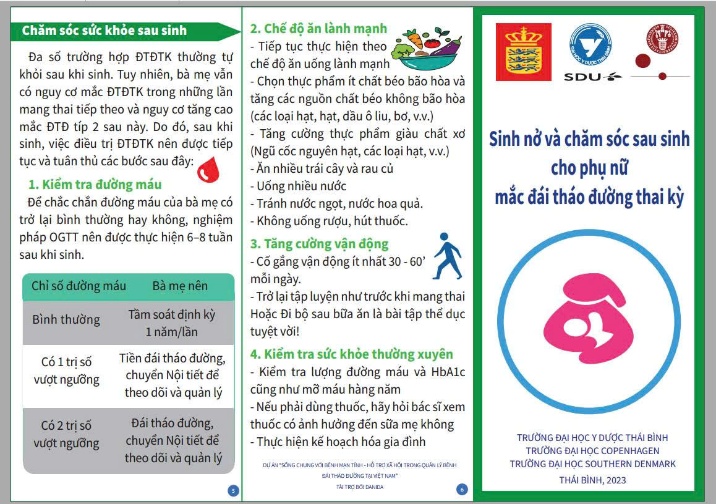** |  | **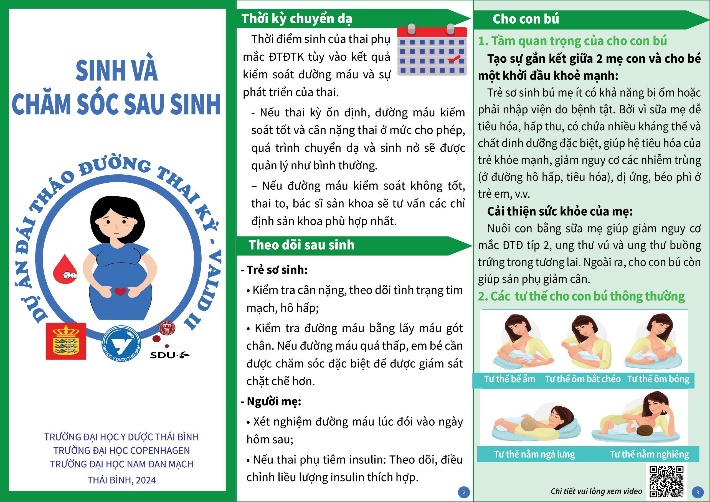** |
|  |  |  |
| **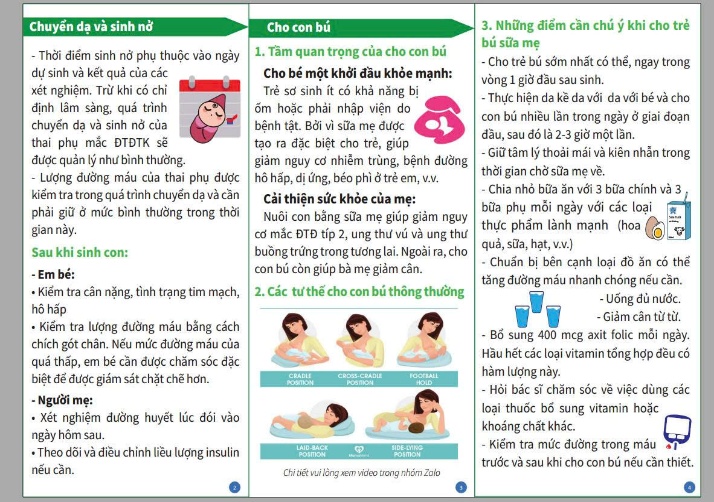** |  | **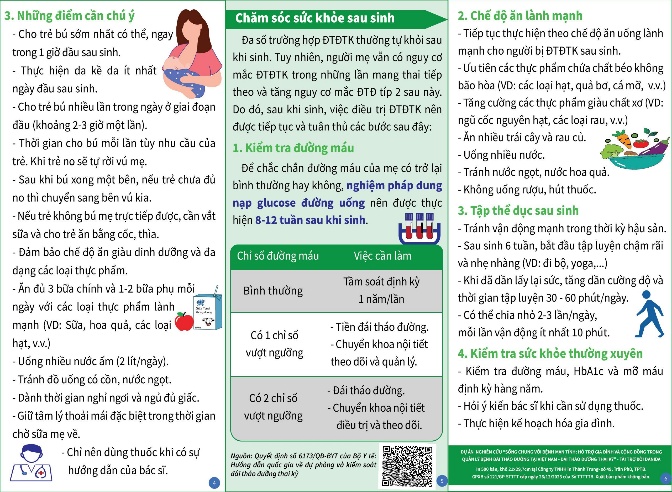** |
