# Supplementary material for: Digital Health–Based Peer Support Ecosystem for Gestational Diabetes Mellitus in Vietnam (VALID II Study): Multistakeholder Cocreation and Pilot Study
Source: J Med Internet Res. 2026 May 6;28:e82434. doi: 10.2196/82434 (PMC13148331; doi:10.2196/82434)
Supplement: Multimedia Appendix 3 [file jmir-v28-e82434-s003.docx]

# EVALUATION OF EDUCATIONAL MATERIALS

Project: "Living Together with Chronic Disease: Informal Support for Diabetes Management in Vietnam, Phase II: Gestational Diabetes in Vietnam (VALID II).”

Hello Madam!

My name is ………………................................ and I am currently participating in the research project: "Living Together with Chronic Disease: Informal Support for Diabetes Management in Vietnam, Phase II: Gestational Diabetes in Vietnam.” This study was conducted in collaboration with Thai Binh University of Medicine and Pharmacy and two universities in Denmark (University of Copenhagen and University of Southern Denmark).

In this project, we will provide you with a set of educational materials (including a leaflet and video) containing essential knowledge and practical guidance to help pregnant women with GDM have a healthy pregnancy and safe childbirth.

We hope this project can help pregnant women share difficulties and challenges in self-care and GDM management. Therefore, we would appreciate your opinions, feedback, and suggestions to help us improve the educational materials to be more engaging and accessible to pregnant women with GDM.

Full name (optional): .........................................................

Year of birth: .........................................................

Address: .........................................................

Number of pregnancies: ………………. – GDM diagnosis number: ………………

The following questions refer to your impressions of the GDM leaflet/video that you have read/watched. Please review the leaflet/video and rate it based on your impressions.

Please circle a number on the scale from 1 to 10 that best reflects your opinion.

| **No.** | **Question** | **Rating Scale** | | | | | | | | | |  |
| --- | --- | --- | --- | --- | --- | --- | --- | --- | --- | --- | --- | --- |
|  | How do you rate the **use of language** in the GDM leaflet? | 1 | 2 | 3 | 4 | 5 | 6 | 7 | 8 | 9 | 10 |  |
|  |  | 1: Very difficult to understand  10: Very easy to understand | | | | | | | | | |  |
|  | How do you rate the **font size** used in the leaflet? | 1 | 2 | 3 | 4 | 5 | 6 | 7 | 8 | 9 | 10 |  |
|  |  | 1: Very difficult to read  10: Very easy to read | | | | | | | | | |  |
|  | How do you rate the **illustrations** in the leaflet? | 1 | 2 | 3 | 4 | 5 | 6 | 7 | 8 | 9 | 10 |  |
|  |  | 1: Not interesting or engaging at all  10: Very interesting and engaging | | | | | | | | | |  |
|  | How do you rate the **information content** in the leaflet? | 1 | 2 | 3 | 4 | 5 | 6 | 7 | 8 | 9 | 10 |  |
|  |  | 1: Very boring  10: Very useful | | | | | | | | | |  |
|  | Please share any comments or suggestions you have to help us improve the quality of the leaflet:  ____________________________________________________________________________________  ____________________________________________________________________________________  ____________________________________________________________________________________  ____________________________________________________________________________________  ____________________________________________________________________________________  ____________________________________________________________________________________  ____________________________________________________________________________________  ____________________________________________________________________________________  ____________________________________________________________________________________  ____________________________________________________________________________________  ____________________________________________________________________________________ | | | | | | | | | | | |
