# Supplementary material for: Digital Health–Based Peer Support Ecosystem for Gestational Diabetes Mellitus in Vietnam (VALID II Study): Multistakeholder Cocreation and Pilot Study
Source: J Med Internet Res. 2026 May 6;28:e82434. doi: 10.2196/82434 (PMC13148331; doi:10.2196/82434)
Supplement: Multimedia Appendix 4 [file jmir-v28-e82434-s004.docx]

**Multimedia Appendix 4. Overview of educational materials**

**Table S1. Overview of co-created GDM educational leaflets and videos: Thái Bình, Vietnam (2023–2024).**

| **No** | **Topic** | **Format** | **Place/Platform** |
| --- | --- | --- | --- |
| 1 | GDM – Things to know | 01 printed leaflet  01 video | Thái Bình Maternity Hospital/  Kim Ngan Clinic  Zalo groups, Private Facebook group |
| 2 | Nutrition therapy | 01 digital leaflet  01 video | Zalo groups, Private Facebook group |
| 3 | Serving and portion guide | 01 digital leaflet  01 video | Zalo groups, Private Facebook group |
| 4 | Physical activities - Prenatal yoga | 01 digital leaflet  02 videos | Zalo groups, Private Facebook group |
| 5 | SMBG at home | 01 digital leaflet  01 video | Zalo groups, Private Facebook group |
| 6 | Family support | 01 digital leaflet | Zalo groups, Private Facebook group |
| 7 | Mental health - Meditation and breathing | 01 digital leaflet  01 video | Zalo groups, Private Facebook group |
| 8 | Childbirth and postpartum care | 01 digital leaflet  01 video | Zalo groups, Private Facebook group |
