# Supplementary material for: Digital Health–Based Peer Support Ecosystem for Gestational Diabetes Mellitus in Vietnam (VALID II Study): Multistakeholder Cocreation and Pilot Study
Source: J Med Internet Res. 2026 May 6;28:e82434. doi: 10.2196/82434 (PMC13148331; doi:10.2196/82434)
Supplement: Multimedia Appendix 5 [file jmir-v28-e82434-s005.docx]

# **Multimedia Appendix 5. System schematic and information flow at TBUMP.**

## **Table S1. Staff for the “Healthy Pregnancy” digital health-based peer support model.**

| **Component** | **Responsibilities** |
| --- | --- |
| **General management** | |
| **Administrator** | Manage Zalo group, post content, coordinate members, and plan Zoom sessions |
| **Observer** | Track participation, summarise feedback, and propose adjustments |
| **Peer support group** | |
| **Active members** | Share experiences, model self-care, and encourage others |
| **HCPs** | Provide advice, correct misinformation, and support peers |
| **Online consulting (Zoom)** | |
| **Endocrinologist** | Explain GDM, answer questions, promote adherence, and build confidence |
| **Nutritionist** | Provide guidance on diet, answer food-related questions, and adjust menus by pregnancy stage and GDM levels |
| **Obstetrician** | Provide prenatal guidance, delivery planning, and breastfeeding support |

## **Operational workflow and triage logic for the "Healthy Pregnancy" GDM intervention**


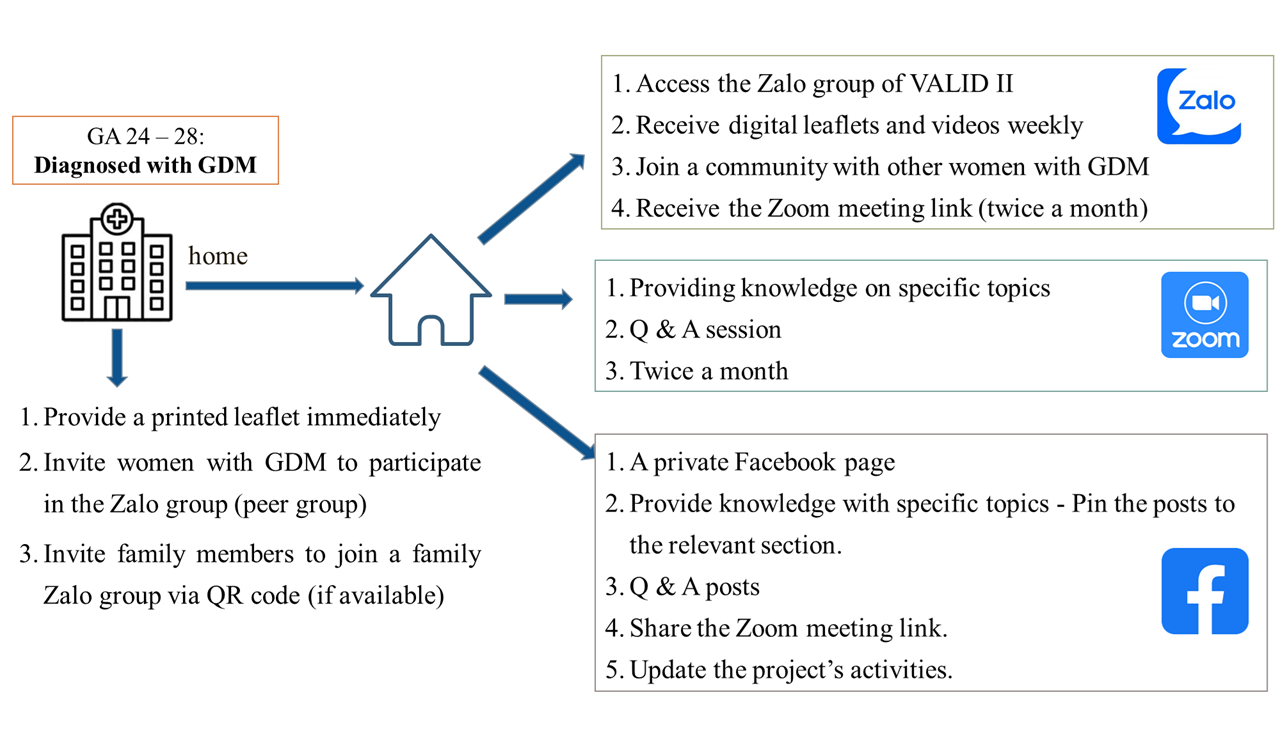


### **Figure S1. Operational workflow and triage logic for the "Healthy Pregnancy" GDM intervention (2024).**

***Upon diagnosis with GDM:***

- The pregnant woman was informed about the intervention's purposes and structure.
- She was invited to participate in the intervention program by entering a Zalo group via the QR code.
- A new QR code for a new Zalo group was launched whenever a group reached approximately 20 participants.

***In the Zalo group:***

- Each participant received "welcoming messages" and a set of GDM self-care leaflets and videos
- They were notified about a specific self-care topic weekly and encouraged to pose questions and comments or mutually share experiences with the other women daily.
- Active members: 1-2 pregnant women per group, who seemed particularly interested in GDM and were more active, were invited to participate as active members. These members received a monthly allowance of $ 7.50 per person.
- If questions regarding emergencies or complex issues were left unanswered or incorrectly answered, an HCP answered, adding to the knowledge bases provided by the leaflets, videos, and other group members' comments.

Each Zalo group remained active until 4 months postpartum.

### **Flow of peer and professional support groups on Zalo group:**

|  | 1. **Group formation:**  - Pregnant women with similar gestational weeks. | |
| --- | --- | --- |
|  |  |  |
|  | 1. **New member onboarding:**  - New members join via QR code - Administrator sends a friendly welcome message and educational materials (leaflets/videos) | |
|  |  | |
|  | 1. **Weekly topic discussion:**  - Administrator sends specific topics or topics based on the group’s needs - Active members encourage discussion and sharing | |
|  |  | |
|  | 1. **Handling medical questions/incorrect information:**  - HCW provides information/correction using friendly, accessible language - Encourages members to continue sharing | |
|  |  | |
|  | 1. **Continuous interaction promotion:**  - Administrator and active members continuously promote interaction within the group | |
|  |  | |
|  | 1. **Regular monitoring and recording:**  - Observer periodically records indicators: number of questions, interactions, discussion content, and friendliness level | |

### **The potential scenarios and recommended responses in Zalo group**


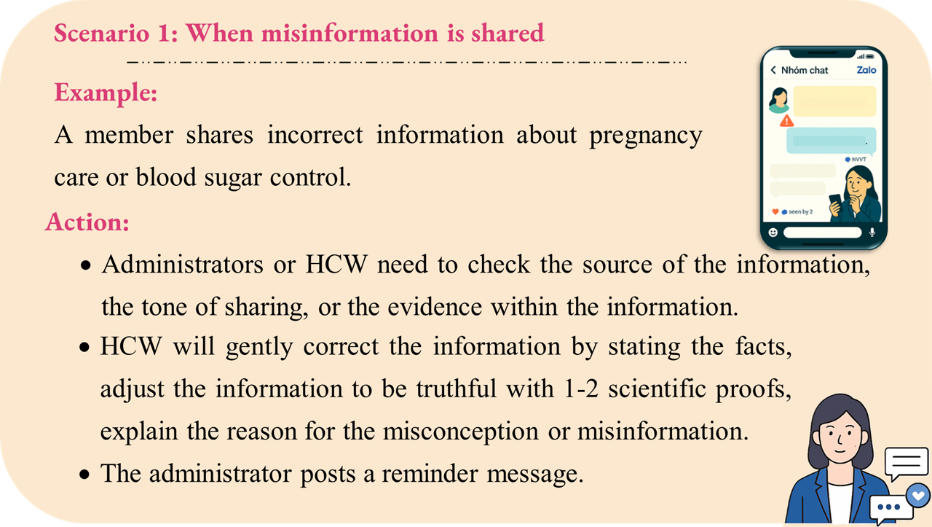
Various situations and unexpected issues may arise during the implementation of the intervention model. The following section presents potential scenarios and recommended responses:


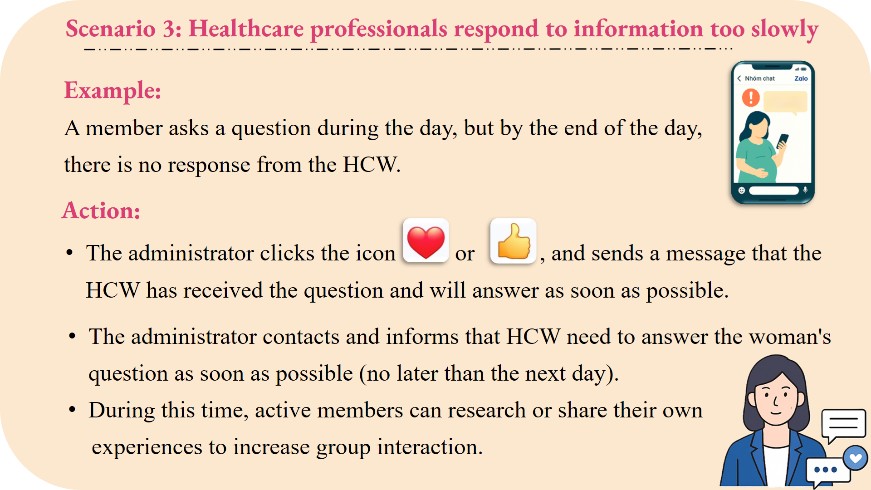

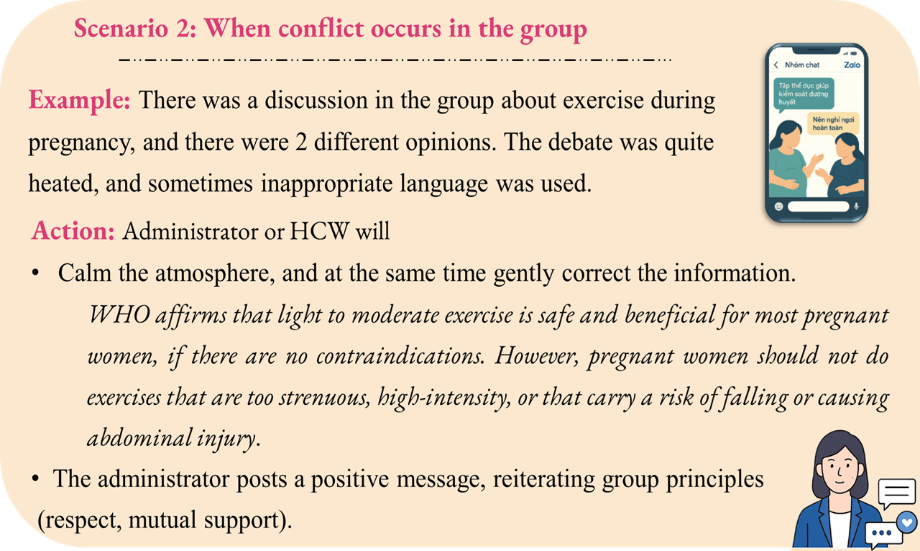


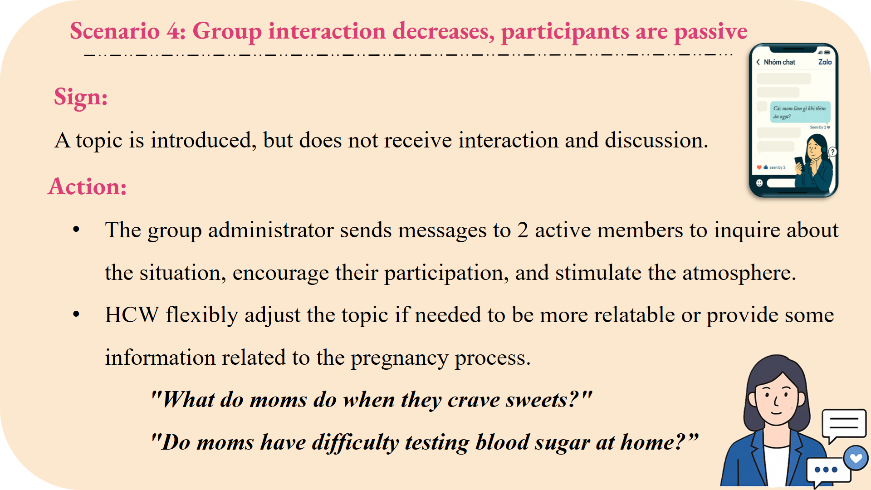


### **Flow of the online consulting group via Zoom meetings, which are held every second Friday**

- Before the Zoom meetings:
- Send Zoom session notification 2 days in advance and reminders 1 day & 1 hour in advance (Sample messages)
- Collect questions from the members to send to specialists
- Provide technical support for pregnant women unfamiliar with Zoom, with photos/videos of Zoom instructions, or direct support
- During the Zoom meetings:
   *Participants*
- Specialist in the relevant field under discussion;
- Pregnant women with GDM in the Zalo group;
- Their family members (optional);
- 1 administrator and 1 observer.

*Zoom meeting process*

### **Table S2. Zoom meeting process (40 minutes per section).**

| Activities | Duration | Notes |
| --- | --- | --- |
| Welcome session; introduction of the specialist & the topic.  Inviting pregnant women to introduce themselves | **5 mins** | Pregnant women could invite family members to join the Zoom session |
| The specialist presents the key points that pregnant women should be aware of | **5 – 10 mins** | Avoid passive lecturing, encourage engagement |
| Question-and-answer session between the specialist and pregnant women | **15 – 20 mins** | Actively encourage participants to share experiences and ask questions throughout the session. |
| Ending | **5 mins** | Thank the pregnant women and the specialist for their participation  Notify about the upcoming Zalo group post with the date & topic  Wish participants a pleasant evening |

- After the Zoom meetings: Post a brief summary in the Zalo group, highlighting key messages, thank the pregnant woman for their participation, and announce the next session.

### **Official Facebook page:**

- Establish an official Facebook page to disseminate topic-specific information related to GDM
- Regularly update program activities, Zoom meeting links, and Q&A content

## **Sample messages used in Zalo groups**

### **Table S3. Sample messages for Zalo group moderators.**

| Sample messages | Attached materials |
| --- | --- |
| Welcome to the "Healthy Pregnancy" intervention program – part of the Gestational Diabetes project in Vietnam! By joining us, you’ll receive helpful tips and knowledge through brochures, videos, and weekly Zoom meetings. Please don't hesitate to ask us any questions along the way. Thank you for being here with us! | Leaflets and videos |
| We understand you might be feeling a bit anxious about your baby’s and your health after a GDM diagnosis. Please take a look at our brochure and video: "Gestational Diabetes Mellitus – What you need to know" for peace of mind. | Leaflet and video: "Gestational Diabetes Mellitus – What you need to know." |
| Hello! Wishing you a wonderful day! Keeping a happy mind during pregnancy is the best gift for your baby's development. Take a deep breath, stay positive, and make time for yourself—whether it’s light exercise, yoga, meditation, or just listening to music. You can also chat with other moms to share your experiences and connect. We invite you to join our meditation and breathing session with our Yoga coach! Sending you health and joy! | Leaflet: " Mental Health" & Video: "Meditation and Breathing" |
| Wishing you a day full of joy! Gestational Diabetes usually appears around weeks 24-28. While it can lead to complications, the good news is you can prevent them by adjusting your diet and staying active. Thank you for reading! Best regards from the VALID II Team. |  |
| Hello! You’ve likely spent the past week getting used to managing GDM and may have many questions. We’d love for you to join our Zoom meeting with an Endocrinology Expert to get more helpful guidance. | Zoom info: Time, Link, Meeting ID, Passcode |
| Wishing you a happy new day! Good nutrition is the foundation for a healthy baby. For moms with GDM, it’s even more vital for stable blood sugar. Select the right foods to support your health and your baby's development. Watch the video below for detailed nutrition tips! | Leaflet and video: "Nutrition therapy." |
| Hello! Wishing you a great day! Try to maintain a balanced diet that includes all food groups: carbohydrates, protein, healthy fats, vitamins, and minerals. It’s best to avoid sugary treats (such as dried fruit or cake) and opt for several small meals a day rather than a few large ones. Check out our brochure and video on Healthy Eating for GDM! | Leaflet and video: "Serving and Portion Guide." |
| Hello! After your diagnosis, you might feel a bit overwhelmed by your new meal plan. Join our Zoom session with a Nutritionist for expert tips—especially on how to enjoy the upcoming Tet holidays while staying healthy! | Zoom info: Time, Link, Meeting ID, Passcode |
| Hello! Wishing you a joyful day! Your family is your biggest support system. To help your loved ones better understand GDM, we’ve created a Zalo group specifically for family members. Please invite them to scan the QR code below to join! | QR code for Family Zalo Group |
| The VALID II Project wishes you and your family a Happy New Year filled with health, success, and happiness. Thank you for being part of our project! | Lunar New Year Greeting message |
| Wishing you a wonderful day! Staying active is a cornerstone of GDM treatment. It helps keep your blood sugar under control and is a healthy choice for both you and your baby. You can find many exercise examples in our brochure. Thank you for your time! | Leaflet and video: "Physical Activity." |
| Practising prenatal yoga can offer numerous benefits to both your physical and mental health. We invite you to follow along with our Prenatal Yoga video, led by our expert coach! | Video: "Prenatal Yoga" |
| Hello! Physical activity is one of the key factors in stabilising your blood sugar levels. Join our Zoom session with an Endocrinology Expert to learn more helpful tips! | Zoom info: Time, Link, Meeting ID, Passcode |
| Hello! Wishing you a happy day! Managing GDM well reduces risks for both you and your baby. One of the best ways to stay in control is by checking your blood sugar regularly. You can use a home monitor or visit a clinic. Check out the brochure and video below for more details! | Leaflet and video: "Self-monitoring blood glucose at home." |
| Hello! Monitoring your sugar at home helps you take charge of your health. Join our Zoom session with an Endocrinology Expert to learn how to do it effectively! | Zoom info: Time, Link, Meeting ID, Passcode |
| Hello! Remember, you are not alone on this journey. Pregnancy is sacred and needs the support of family and community. Please share your feelings and challenges with those around you or with other moms in the VALID II Zalo group. Don't hesitate to ask for help when you need it! | Leaflet: "Family Support" |
| Hello! We know you’re starting to pack your bags to welcome your little angel. Join our Zoom session with an Obstetrician to learn everything you need to know about labour and delivery! | Zoom info: Time, Link, Meeting ID, Passcode |
| Hello! Wishing you a joyful day! As you know, GDM affects both mom and baby. Besides lifestyle changes, checking blood sugar for both of you during delivery is very important. Please notify the VALID II research team when you head to the hospital so we can best support you. Wishing you a safe and smooth delivery! |  |
| Hello! Wishing you a great start to the week! Breast milk is the best nutrition for babies under 6 months, filled with natural antibodies. Exclusive breastfeeding for the first 6 months helps your baby grow strong. If you have any breastfeeding concerns, watch our video or share your experience in our Zalo group! | Leaflet: "Delivery and Postpartum Care" |
| Hi everyone! Has anyone looked into the long-term risks of GDM, like Type 2 diabetes after giving birth? In this Friday's Zoom session, our Obstetrician will share important notes for both before and after delivery. See you there! | Zoom info: Time, Link, Meeting ID, Passcode |
| Hello! Wishing you a great day! Women who have had GDM have a higher risk of Type 2 diabetes later on. Because of this, please ensure that you have a 2-hour Oral Glucose Tolerance Test between 6 and 12 weeks after your baby is born. This is our final message—thank you so much for being with us! | Leaflet and video: "Birth and Postpartum Care" |
